# Chemotherapy in NEN: still has a role?

**DOI:** 10.1007/s11154-021-09638-0

**Published:** 2021-04-11

**Authors:** Paula Espinosa-Olarte, Anna La Salvia, Maria C. Riesco-Martinez, Beatriz Anton-Pascual, Rocio Garcia-Carbonero

**Affiliations:** Oncology Department, Hospital Universitario, 12 de Octubre, Imas12, UCM, Madrid, Spain

## Abstract

Neuroendocrine neoplasms (NENs) comprise a broad spectrum of tumors with widely variable biological and clinical behavior. Primary tumor site, extent of disease, tumor differentiation and expression of so matostatin receptors, proliferation and growth rates are the major prognostic factors that determine the therapeutic strategy. Treatment options for advanced disease have considerably expanded in recent years, particularly for well differentiated tumors (NETs). Novel drugs approved over the past decade in this context include somatostatin analogues and ^177^Lu-oxodotreotide for somatostatin-receptor-positive gastroenteropancreatic (GEP) NETs, sunitinib for pancreatic NETs (P-NETs), and everolimus for P-NETs and non-functioning lung or gastrointestinal NETs. Nevertheless, chemotherapy remains an essential component of the treatment armamentarium of patients with NENs, particularly of patients with P-NETs or those with bulky, symptomatic or rapidly progressive tumors (generally G3 or high-G2 NENs). In this manuscript we will comprehensively review available evidence related to the use of chemotherapy in lung and GEP NENs and will critically discuss its role in the treatment algorithm of this family of neoplasms.

## Introduction

Neuroendocrine neoplasias (NENs) are a heterogeneous family of malignancies of wide anatomic distribution, as they arise from neuroendocrine cells distributed throughout the body forming glands or as part of the diffuse neuroendocrine system. The majority of NENs are from gastroenteropancreatic (GEP) (66%) and bronchopulmonary origin (31%), although they may develop in any organ. Their incidence has significantly increased over the past 4 decades, from 1,09 (1973) to 6,98 (2012) new cases per 100.000 inhabitants per year, as well as their prevalence (from 0.006% (1993) to 0.048% (2012) 20-year limited-duration prevalence) [1]. The raise in incidence has been observed across all tumor sites, stages and grades, likely due to improved diagnostic techniques and greater clinical awareness. Survival has also improved over time as a consequence of earlier detection and the therapeutic advances achieved over the past decades [1]. GEP-NENs are classified based on morphology and proliferation rate in well differentiated neuroendocrine tumors (NETs) (G1 to G3) or poorly differentiated large or small cell neuroendocrine carcinomas (NECs) (all G3) [2]. Lung-NENs are classified based on morphology and mitotic count as well differentiated typical or atypical carcinoids, or poorly differentiated large or small cell NECs [3]. Tumor differentiation in both GEP and lung NENs reflect two major biologically and genetically distinct entities with very different clinical behavior, including response to available treatments. Primary tumor site, extent of disease, tumor expression of somatostatin receptors, proliferation and growth rates are also major prognostic factors that shall be taken into account to define treatment strategy.

Treatment options for advanced disease have considerably expanded in recent years, particularly for NETs [4]. Most remarkable drugs recently incorporated to the treatment armamentarium include somatostatin analogues (lanreotide, octreotide) for G1 or low G2 gastroenteropancreatic (GEP) NETs [5, 6], sunitinib for pancreatic NETs (P-NETs) [7], everolimus for non-functioning lung or gastrointestinal NETs (L-or GI-NETs) and P-NETs [8, 9], and ^177^Lu-oxodotreotide for somatostatin-receptor-positive GEP-NETs [10]. Despite these unquestionable steps forward, options are still rather limited, and chemotherapy remains an essential component of the treatment strategy of patients with NENs, particularly for those with bulky, symptomatic or rapidly progressive tumors (generally G3 or high-G2 NENs). In the setting of well differentiated NENs, chemotherapy is primarily indicated in tumors of pancreatic origin (P-NETs), whereas its use in L- or GI-NETs shall be reserved for selected patients who have failed other more effective therapeutic options.

**Fig. 1:**
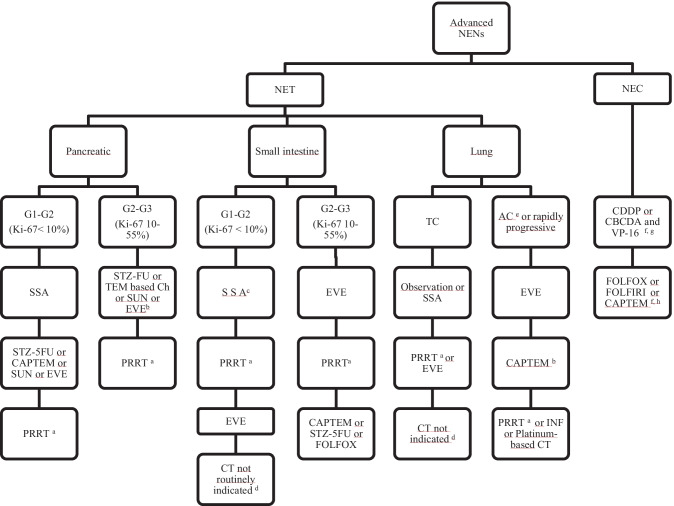
Treatment algorithm of advanced NENs. AC, atypical carcinoid; CAPTEM, capecitabine-temozolomide; CDDP, cisplatin; CBCDA, carboplatin; CT, chemotherapy; EVE, everolimus; FOLFIRI, 5-fluorouracil and irinotecan; FOLFOX, 5-fluorouracil and oxaliplatin; INF, interferon-alfa; NENs, neuroendocrine neoplasias; NET, neuroendocrine tumor; NEC, neuroendocrine carcinoma; PRRT, peptide receptor radionucleotide therapy; SSA, somatostatine analogues; STZ-5FU, streptozocin-5 Fluorouracile; SUN, sunitinib;TC, typical carcinoid; VP-16, etoposide. ^a^In somatostatin-receptor imaging positive tumors and/or refractory hormonal síndrome. ^b^Chemotherapy preferred upfront over targeted agents in G3 NETs. ^c^Watch and wait may be considered in G1 very indolent tumors, particularly in older or frail patients. ^d^CAPTEM may be considered after progression to all available treatments in selected patients with good PS and rapidly progressing tumors. ^e^Chemotherapy may be considered upfront in selected patients (rapidly progressing tumors, Ki-67>20%).^f^Enrollement in clinical trials is recommended if available.^g^Carboplatin is preferred over cisplatin due to its more favorable toxicity profile.^h^The treatment choice should be based on response to prior therapy, toxicity profile, residual toxicity from prior chemotherapy (i.e. neurotoxicity) and patient’s comorbidities and preferences (i.e. oral vs iv)

In this manuscript we will critically review available evidence for the use of chemotherapy in lung and GEP NENs and will discuss its current role in the treatment algorithm of this family of neoplasms (Fig. 1).

### Role of chemotherapy in neuroendocrine tumors

Chemotherapy in NENs initially focused on the treatment of islet cell carcinomas, that is P-NETs per current terminology, following the discovery of the alkylating agent streptozotocin (STZ), a glucose analogue isolated from *Streptomyces achromogenes* that was found to be particularly toxic to the β-cell of the pancreas via GLUT2 transporter uptake. The first randomized trials date from the 80 s’ and provided the first evidence of drug antitumor activity in NETs, leading to FDA-approval of STZ for the treatment of advanced islet cell carcinoma. This is the first drug and solely cytotoxic agent ever approved in the field of NENs. Subsequent trials tested different classical cytotoxic regimens, mainly based in alkylating agents, antimetabolites and anthracyclines. These trials globally demonstrated greater efficacy of chemotherapy in P-NETs than in NETs from lung or GI origin. However, results of these trials are not easy to place in the context of current standards of care, as methods to assess response were less rigorous and standardized, tumor classification was exclusively based on morphology and did not address relevant prognostic features such as the proliferation rate, and study populations were often underpowered and included a wide range of non-stratified primary tumor sites, except for those specifically devoted to P-NETs. Consequently, evidence will be discussed within two major subgroups, P-NETs and extrapancreatic-NETs, as the design of most trials does not allow to extract specific recommendations for lung or different GI tumor sites.

### Pancreatic NETs

Streptozocin (STZ) is an alkylating agent that was the first drug to demonstrate efficacy in NETs. A seminal randomized trial published by Moertel et al. in 1980 assessed STZ alone and in combination with 5-fluorouracil (5-FU) in patients with advanced islet cell carcinomas, where the combination showed significantly greater response rates (RR), 63 vs. 36%, and a tendency to an improved survival (26 vs. 16 months) [11]. A second randomized phase III trial also conducted in patients with advanced P-NETs showed improved outcomes with the combination of STZ and doxorubicin (DOXO) as compared to STZ and 5-FU in terms of RR (69 vs. 45%), time to tumor progression and overall survival (OS) (26 vs 17 months) [12]. Despite its superior efficacy, however, the use of the STZ-DOXO combination has been limited by its less favorable toxicity profile, particularly due to DOXO-induced cumulative cardiotoxicity and alopecia. It should be noted that RR in these trials were not assessed by current standardized RECIST criteria; they included clinical, biochemical and radiological responses, and are therefore not comparable to objective radiological response (ORR) reported in recent trials. Three-drug combinations such as STZ, capecitabine and cisplatin (CDDP) did not demonstrate greater efficacy compared to STZ-capecitabine doublet regimens while they were significantly more toxic [13]. The STZ-5-FU regimen has been also assessed in combination with bevacizumab, a humanized monoclonal antibody targeting the vascular endothelial growth factor (VEGF), in the phase II BETTER trial that included 34 patients with P-NETs. The disease control rate (DCR) confirmed by centralized review was 100%: 19 patients (56%) achieved a partial response and 15 (44%) had stable disease as best response. Median PFS was 23,7 months and the OS rate at 2 years was 88% [14].

Dacarbazine (DTIC) is a synthetic alkylator that must be metabolized by cytochrome P450 (CYP450) to form its active metabolite, 3-methyl-(triazen-1-yl)imidazole-4-carboxamide (MTIC). The first prospective trial with DTIC at high doses in P-NETs was conducted by Ramanathan et al. [15] and reported an ORR of 33% (50% among the 28 chemonaïve patients), and a median OS of 19,3 months. Toxicity was not negligible, with G3-4 adverse events (AEs) documented in 30% of patients (mainly vomiting and hematological toxicity) and 2 toxic deaths (one septic shock and one myocardial infarction). Safety is improved at lower doses as seen in subsequent trials, and it should also be noted that supportive care for emesis and myelotoxicity has substantially improved since these trials were conducted. DTIC has also been tested in small prospective non-randomized trials in combination with 5-FU and epirubicin with ORRs (25–27%) that did not seem to be greater than DTIC monotherapy, whereas the combination substantially increases toxicity [15–17].

Temozolomide (TEM) is an oral derivative of DTIC which has displaced it in the treatment of NETs. It is also a prodrug that is spontaneously hydrolyzed at physiological pH to MTIC. MTIC methylates DNA commonly at the N-7 or O-6 positions of guanine residues. This DNA damage is repaired by the O-6-methylguanine-DNA methyltransferase (MGMT) and epigenetic silencing of the MGMT gene render cells more sensitive to this agent. MGMT expression loss assessed by immunohistochemistry or MGMT promoter methylation assessed by PCR or pyrosequencing has been successfully used as a positive predictive biomarker for TEM efficacy in other solid tumors, although its value in NETs is controversial [18, 19]. Other DNA repair systems involved in reverting TEM-induced DNA damage include the mismatch repair system and poly(ADP)-ribose polymerase (PARP) pathway, and tumors with defects in these pathways could potentially be more sensitive to TEM. TEM crosses the blood–brain barrier and its toxicity profile (mainly nausea, vomiting, fatigue and hematological toxicity) is easily manageable.

TEM was first explored in some small prospective studies in combination with different targeted agents including antiangiogenics (thalidomide, bevacizumab) and mTOR inhibitors (everolimus), that reported ORR of 30–45% [20–22]. Retrospective studies also suggested promising activity for the combination of TEM and capecitabine (CAP) with response rates of up to 70% [19, 23–30]. But the most solid evidence of TEM efficacy in P-NETs has been recently provided by the E2211 randomized phase II trial conducted by the ECOG-ACRIN Cancer Research Group [31]. This trial included 144 patients with advanced G1-2 P-NETs that were randomized to receive TEM alone or in combination with CAP (CAPTEM). Prior permitted therapies included somatostatin analogues (received by 53–54% of patients), everolimus (35–36%) or sunitinib (11–13%), but not chemotherapy. ORR was not significantly different among study arms (33 vs. 28%), but CAPTEM demonstrated improved PFS (22,7 vs. 14,1 months, HR 0.58, P = 0.023) and OS (not reached vs. 38 months, HR 0.41, P = 0.012) as compared to TEM monotherapy. Of note, a greater proportion of G2 tumors was observed in the TEM arm (55 vs. 32%), although grade was not significantly associated with PFS nor OS, and the benefit in survival favoring CAPTEM was still significant after adjustment for grade. G3-4 AEs were more commonly encountered in the CAPTEM arm (44 vs. 22%, P = 0.007). Significantly increased treatment related G3-4 AEs in the CAPTEM arm were neutropenia (13 vs. 4%), nausea (8 vs. 0%), vomiting (8 vs. 0%), diarrhea (8 vs. 0%) and fatigue (8 vs. 1%). Treatment in the E2211 trial was continued until disease progression or unacceptable toxicity to a maximum of 13 cycles, although the optimal treatment duration is a matter of debate [32, 33].

CAP and TEM are both radiosensitizer drugs that have been used to increase the activity of peptide receptor radionuclide therapy (PRRT). The combination demonstrated to be safe in advanced NET patients in a phase I/II trial [34]. Promising results have been reported for the combination of CAPTEM with ^177^-Lu-Octreotate in a prospective study, showing an ORR of 80% in P-NETs [35]. Based on these encouraging results, the Australasian Gastrointestinal Trials Group (AGITG) designed the CONTROL NET Study, a Phase II randomized (2:1) exploratory study evaluating the activity of ^177^Lu-Octreotate peptide receptor radionuclide therapy (PRRT) and CAPTEM in 2 patient cohorts. The P-NET cohort was randomized (2:1) to receive PRRT and CAPTEM vs CAPTEM alone, and the midgut cohort was randomized to receive PRRT and CAPTEM vs PRRT alone. Preliminary results recently presented of the P-NET cohort (N = 27) showed numerically higher ORR for the combination (67 vs. 33%) with no clear PFS benefit (PFS rate at 1 year: 76 vs. 67%), and at the expense of greater G3-4 toxicity, mainly hematological. Longer follow-up to 36 months is planned to see whether a PFS benefit is observed sufficient to warrant phase III evaluation. Further follow-up is also required to carefully assess bone marrow toxicity, specifically regarding the eventual development of myelodysplastic syndrome or acute myeloid leukemia, which may be notably increased when PRRT is administered with alkylating agents and is of particular concern [36]. A phase I trial has explored the combination of TEM with TAS102. TAS102 consists of trifluridine, a nucleoside analog, and tipiracil, a thymidine phosphorylase inhibitor that prevents rapid metabolism of trifluridine, increasing thereby its bioavailability. TAS102 is non-cross resistant with 5-fluorouracil and capecitabine and has a different toxicity profile. In this trial the combination was well tolerated and the most frequent EAs ≥ G3 were hematological (33% neutropenia, 27% lymphopenia, 27% thrombocytopenia. The 8% of evaluable patients [13] had partial response, and the disease control rate (DCR) was 92%. Enrollment into the expansion cohort of patients with advanced G1-2 P-NETs is ongoing [37].

Other fluoropyrimidine-based regimens explored in P-NETs include different combinations with irinotecan or oxaliplatin. The FOLFIRI regimen (irinotecan and 5-FU as continuous infusion), widely used in colorectal cancer and other digestive malignancies, has been tested in a prospective French study that included 20 chemo-naïve P-NETs and reported an 80% progression-free rate at 6 months (primary endpoint). Nevertheless, only one partial response was observed and this regimen was not exempt of toxicity, with G3-4 adverse events observed in 80% of patients [38]. A phase II trial by Ducreux et al. that included a small number of pretreated P-NET patients (N = 10) also reported limited efficacy of FOLFIRI (ORR of 10%) [39]. Oxaliplatin-based regimens such as CAPOX, FOLFOX or GEMOX (Gemcitabine and oxaliplatin), have also demonstrated to be active in advanced NETs, even though they have been generally assessed as a second line treatment. The largest retrospective study of 78 patients with NETs from different origins treated with CAPOX, GEMOX or FOLFOX, including 36 P-NETs with a mean ki-67 index of 14,4%, globally reported an ORR of 33% [40]. Prospective trials with platinum-based therapy are scarce and include a small number of NETs of pancreatic origin [41–44]. The phase II trial of Bajetta et al. [43] included 40 NEN patients, 11 with P-NETs, that were treated with XELOX. The ORR for P-NETs was 27%. Of note, in this trial the ORR was superior in NETs (30%) than in NECs (23%). The combination of FOLFOX with Bevacizumab and CAPOX with bevacizumab was tested in two phase II trials. 12 and 16 patients with P-NETs were included, showing an ORRs of 50% and 19%, respectively. About 90% of patients recruited in both trials were well differentiated tumors (NETs), and the combination showed an overall disease control rate (DCR) of 94% with FOLFOX-Bevacizumab and of 78% with CAPOX-Bevacizumab [44]. Regarding chemotherapy and antiangiogenic regimens, a novel interesting approach recently explored the administration of sunitinib (SUN), a tyrosine kinase inhibitor targeting VEGFR and PDGFR among others, with the hypoxia-targeting prodrug evofosfamide (EVO), formally called TH-302, that is a DNA alkylator selectively activated under hypoxic conditions the SUNEVO trial (GETNE-1408) [45]. This study included 17 chemonaïve G1-2 P-NETs. The study reached the planned threshold for efficacy in the first step of simon design, with an ORR of 18%, but was terminated early due to excess toxicity (65% developed treatment-related G3-4 AEs, mostly fatigue and neutropenia; 100% of patients required EVO dose reduction due to toxicity) and also as Merck decided not to continue EVO clinical development following negative pivotal studies in other tumors (pancreatic adenocarcinoma, soft tissue sarcomas).

In summary, based on available evidence from randomized trials we may conclude that STZ-5FU has demonstrated to be more effective than STZ monotherapy in P-NETs (greater RR and a trend towards improved survival), and STZ-DOXO to be more effective than STZ-FU (greater RR, TTP and OS), although STZ-5FU is most widely used as its toxicity profile is more suitable for long-term treatment. However, it should be noted that STZ has never been tested against placebo or best supportive care (BSC), and therefore its individual contribution to efficacy in this setting is difficult to discriminate. More recently, the ECOG-ACRIN E2211 study has demonstrated that both TEM and CAPTEM are able to achieve significant tumor shrinkage assessed per current standard criteria in one third of patients, which is a relevant endpoint in a subgroup of high-risk P-NETs, and CAPTEM significantly impacted on PFS and OS. This regimen is presently the most widely used as its toxicity profile is more favorable and its oral administration is more convenient for most patients. Nevertheless, as with STZ, TEM has been never tested against placebo or BSC in this setting, and to date no published trial has compared these regimens against each other or against other chemotherapy combinations (FOLFOX, FOLFIRI…). Chemotherapy has not been compared either with targeted agents, so the optimal sequence and integration with other therapeutic strategies is still a matter of debate. Some ongoing trials however are currently assessing these issues. The BETTER-2 trial (NCT03351296) randomized G1-3 P-NET patients to receive STZ–5-FU vs. CAPTEM, with or without Bevacizumab, in a two by two design; its primary endpoint is PFS [46]. Moreover, the SEQTOR study (NCT02246127) [47], comparing EVE with STZ-5FU the OCCLURANDOM trial (NCT02230176) [48], comparing SUN with 177Lu-Oxodotreotide, or the COMPETE study (NCT03049189), [49], comparing EVE with 177Lu-Edotreotide, will further provide relevant information in the near future to help clinicians optimize the sequential use of available treatment options in P-NETs. These and other chemotherapy-based trials in P-NETs [50, 51] are summarized in Table 1.

**Table 1:** Chemotherapy in P-NETs: results from phase III and selected phase II trials

Author	Phase	N total (P-NETs*)	Treatment	ORR (%)	mPFS (months)	mOS (months)
Classical regimens based on STZ, DOXO and DTIC						
Moertel et al 1980 [11]	III	84	STZ + 5-FU vs STZ	63.0 vs 36.0	NR	26.0 vs 16.5 (P = ns)
Moertel et al 1992 [12]	III	105	STZ + DOXO vs STZ + 5-FU vs CTZ	69.0 vs 45.0 vs 30.0 ( P = 0.005)	20.0 vs 6.9 vs NR ^b^ ( P < 0.001)	26.4 vs 16.8 vs 18 (P < 0.004)
Meyer et al 2014 [13]	II	86 (41)	STZ + CAP vs STZ + CAP + CDDP	12 vs. 16 ^a^	10.2 vs 9.7 ^a^ (HR 0.74/P = NR)	26.7 vs 27.5 ^a^ (HR 1.16/P = NR)
Ramanathan RK et al. 2001[15]	II	52	DTIC (high dose)	34.0	NR	19.3
Bajetta E. et al 1998 [17]	II	30 (15)	DTIC + 5-FU + EPI	27.0	NR	Not reached
Bajetta E et al. 2002 [16]	II	82 (28)	DTIC + 5-FU + EPI	24.4 ^a^	21.0 ^a/b^	38.0 ^a^
Ducreux et al 2014 [14]	II	34	BEVA + 5-FU + STZ	52.0	26.3	Not reached
Temozolomide and Capecitabine based regimens						
Pamela L. Kunz et al. 2018 [31]	II	144	CAPTEM vs TEM	33.3 vs 27.8	22.7 vs 14.4 (HR 0.58/P = 0.023)	Not reached vs 38.0 (HR 0.41/P = 0.012)
Pavlakis N et al. 2020 [36]	II	28	CAPTEM vs 177Lu-Octreotate + CAPTEM	33.3 vs 66.7	NR	NR
Claringbold P.G et al. 2016 [35]	II	30	CAPTEM + 177Lu-Octreotate	80.0	48.0	Not reached
Fine et al 2014 [50]	II	28 (11)	CAPTEM	36.0	Not reached	Not reached
Kulke et al 2006 [20]	II	29 (11)	TEM + Thalidomide	45.0	Not reached	Not reached
Chan JA et al 2012 [21]	II	34 (15)	TEM + BEVA	33.0	14.3	41.7
Chan JA et al 2013 [22]	I/II	40	TEM + EVE	40.0	15.4	Not reached
Platinum, 5-FU or other cytotoxic-based regimens						
Moertel et al 1991 [41]	II	45 (14)	CDDP + VP-16	14.0	4.0	15.5
Fjällskog ML et al. 2001 [42]	II	36 (11)	CDDP + VP-16	27.0	NR	13.0 (including 11 NETs + 4 NECs)
Bajetta et al 2007 [43]	II	27 (11)	XELOX	27.3	20.0 ^a/b^	40.0 ^a^
Kunz PL et al 2016 [44]	II	16	XELOX + BEVA	18.8	15.7	38.0
Kunz PL et al 2016 [44]	II	12	FOLFOX + BEVA	50.0	21.0	31.0
Berruti et al 2014 [51]	II	45 (19)	CAP + BEVA + OCT	26.3	14.3	Not reached
Ducreux et al 2006 [39]	II	20 (10)	FOLFIRI	10.0	5.0 ^a^	15.0 ^a^
Brixi-Benmansour et al. 2011[38]	II	20	FOLFIRI	5.0	9.1	NR
Grande et al 2019 [45]	II	17	SUN + EVO	17.6	10.38	Not reached

*BEVA* Bevacizumab, *CAP* capecitabine, *CAPTEM* capecitabine-temozolomide, *CDDP* cisplatin, *CTZ* chlorozotocin, *DOXO* doxorubicin, *DTIC* dacarbazine, *EPI* epirubicin, *EVE* everolimus, *EVO* Evofosfamide, *FOLFIRI* 5-fluorouracile- Irinotecan, *FOLFOX* 5-fluorouracile-oxaliplatin, *m* months, *mOS* median overall survival, *mPFS* median progression free survival, *NETs* neuroendocrine tumors, *NECs* neuroendocrine carcinomas, *NR* not reported, *ns* non signicant, *OCT* octreotide, *ORR* objective response rate, *P-NETs* pancreatic neuroendocrine tumors, *STZ* streptozocin, *SUN*, *TEM* temozolomide; *VP-16* etoposide, *y* years, *XELOX* capecitabine-oxaliplatin

^*^If more histologies included

^a^ In the entire cohort, not specific of P-NETs

^b^*TTP* time to tumor progression

Key messages

- Chemotherapy is primarily indicated in advanced NETs of pancreatic origin, and is recommended as upfront therapy in tumors with high proliferation rates, G2 in the upper range (Ki-67 > 10%) or G3 (Ki-67 > 20%), particularly in patients with bulky or rapidly progressive disease.
- Randomized trials to date have never compared chemotherapy versus placebo or best supportive care or other treatment options (ie. targeted therapy or PRRT), and this limitation should be bared in mind to interpret treatment recommendations with caution.
- STZ has demonstrated efficacy in P-NETs especially when combined with DOXO or 5-FU The STZ-DOX combination is more effective but more toxic than STZ-5FU, particularly regarding cardiotoxicity and alopecia, and thus STZ-5FU is generally preferred by most clinicians and patients.
- More recently, TEM has also demonstrated efficacy in P-NETs, particularly in combination with CAP, and the oral CAPTEM regimen is being increasingly used due to improved patient’s tolerance and convenience.
- Ongoing randomized trials comparing head-to-head both chemotherapy regimens (CAPTEM vs STZ-5FU), or chemotherapy versus targeted agents (i.e. everolimus), or other currently available treatment options to treat P-NETs (i.e. PRRT vs everolimus or sunitinib) shall help elucidate their relative efficacy and the optimal treatment sequence in these patient

### Extra-pancreatic NETs

Chemotherapy plays overall a minor role in the treatment of well-differentiated NETs of non-pancreatic origin. The term extra-pancreatic NETs (EP-NETs) encompass a wide spectrum of tumors that include primarily gastrointestinal (GI) (esophago-gastric, biliary tract, small and large bowel) and lung NETs, but also feochromocytomas-paragangliomas, medullary thyroid carcinomas, gynecological or urologic NETs, NETs of unknown primary and others. They are indeed a very heterogeneous group in terms of molecular background, prognosis, treatment options and likely responsiveness to different therapies, but they have been traditionally gathered together in clinical trials under the general term “carcinoid”. There are very few tumor-site specifically dedicated chemotherapy trials, and therefore site-specific results are generally obtained as sub-analysis of small cohorts of patients with tumors from a particular anatomic origin included in these mixed trials. In this review we will try to provide site-specific information when possible, although this shall be interpreted with great caution as it generally refers to retrospective, unplanned subgroup analysis of small patient subpopulations.

The first randomized trial in carcinoids (EST 3272) was published by Moertel et al. in 1979. This study included 118 patients with metastatic carcinoid tumors of different primary sites (40 small bowel, 10 other GI sites, 7 pancreas, 17 lung and 18 of unknown primary) that were randomly allocated to receive STZ with cyclophosphamide (CTX) or with 5-FU, with a crossover design to single agent 5-FU or CTX upon disease progression [52]. RRs were 33% for patients in the STZ-5FU arm vs 26% for those treated with STZ-CTX. Of note, RR was significantly greater in patients with small bowel carcinoids (44 vs. 37%, respectively), than in patients with lung tumors (29 vs. 0%) or tumors of unknown primary (29 vs. 0%). Two of 11 patients that crossed-over to 5-FU responded, but none of the 8 patients that crossed over to CTX did. There was no significant difference in survival between the two treatment arms, but survival did differ by primary tumor origin: 28.4 months for small bowel, 24.0 months for pancreas, 15.1 months for lung and 9.0 months for unknown primary. Subsequently, the EST 5275 compared the STZ-5FU regimen with DOXO monotherapy in 172 patients with progressive carcinoid tumors (61 small bowel, 18 other GI, 18 lung, 75 other or unknown) [53]. There were no significant differences among study arms in RR (22 vs. 21%) or survival (median of 16 vs. 12 months). Thirty-three patients who failed STZ-5FU crossed over to DOXO achieving a RR of 18%. Thirty-five patients who failed DOXO and crossed over to STZ-5FU achieved a RR of 29%. Both regimens have therefore similar activity in carcinoid tumors and they mainly differ in their toxicity profiles. Based on the results of these 2 trials, the Eastern Cooperative Group (ECOG) designed the E1281 study, a phase II-III trial that randomized 176 patients with advanced carcinoid tumors (43 small bowel, 12 cecum/rectum, 8 pancreas, 22 lung, and 78 other or unknown) to receive STZ-5FU vs DOXO-5FU [54]. Sixty-one patients were treated with DTIC upon disease progression. There were no significant differences in ORR (16 vs. 15.9%) or PFS (5.3 vs. 4.5 months) among study arms, but survival was significantly greater for patients treated with STZ-5FU (24.3 vs. 15.7 months, P = 0.027). Hematologic toxicities were the major treatment-related toxicities for both DOXO-5FU and STZ-5FU, and mild to moderate renal toxicity was reported in 40 (34.8%) of STZ-5FU-treated patients. The RR of crossover DTIC treatment was 8.2%, with a median survival of 11.9 months. The authors conclude that STZ-5FU is the treatment of choice when chemotherapy is judged to be an option for selected patients with carcinoid tumors. Thereafter, a small randomized trial that included 64 patients with advanced carcinoids (including 6 P-NETs and 58 EP-NETs) compared STZ-5FU with INFα-2A. Despite a trend in favor of IFN, there was no significant differences in PFS and OS among study arms, and there was only one partial response documented in the chemotherapy arm [55]. DTIC was also assessed at high and low doses in 56 patients with advanced carcinoids (28 small bowel, 7 lung, 14 unknown primary and 7 other) with an overall RR of 16% (20% at high doses) and a median survival of 20 months [56]. A retrospective study by Turner et al. evaluated the efficacy of STZ-5-FU in combination with CDDP in 79 patients, 33 with EP-NENs. A RR of 25% was reported for non-pancreatic primary sites (vs 38% for P-NENs), but these included both NETs and NECs and RRs by subgroup were not specified [57]. Moreover, the addition of CDDP to STZ-CAP did not demonstrate greater efficacy compared to STZ-CAP in a randomized study that included 86 patients with advanced NETs of pancreatic (N = 41), gastroduodenal (N = 17) or unknown primary (N = 18), while the 3-drug regimen was significantly more toxic [13].

More recently, following success of these agents in the treatment of P-NETs, capecitabine and temozolomide have been explored in EP-NETs with less encouraging results. CAP in monotherapy showed no ORR in the first trial conducted by Medley et al. in EP-NETs [58], but its combination with bevacizumab or bevacizumab and octreotide in the BETTER and XELBEVOC prospective trials, demonstrated an ORR of 12–18% [51, 59]. Despite retrospective studies showed interesting data of TEM in limited numbers of lung NETs (N = 13, 31% achieved a partial response) [60], prospective studies have not confirmed these results. Clinical trials evaluating TEM combinations generally included a small number of EP-NETs (carcinoids) and their results were quite discouraging, with ORR of 7% with TEM-thalidomide [20] and 0% with TEM-bevacizumab combinations [21]. More recently, preliminary results of the ATLANT study that assessed the combination of TEM and lanreotide in 40 lung or thymus NETs reported only 1 partial response (2.5%) and a median PFS of 9.2 months [61].

Regarding the CAPTEM combination evidence in EP-NETs is rather poor. One of the largest retrospective series to date included 65 NET patients treated with CAPTEM; 19 of them (28%) were of non-pancreatic origin (9 lung, 7 GI and 3 unknown primary). ORR seemed lower in EP-NETs (37%) than in P-NETs (48%). Median PFS and OS was 16.1 months and 38.3 months, respectively, with no significant differences between P- and EP-NETs [30]. Two small retrospective series that included 20–33 patients with lung NETs treated with CAPTEM reported an ORR of 18–30%, a median PFS of 9–13 months and a median OS of 30–68 months [62]. A phase II trial evaluated this combination in a heterogeneous patient population with NETs. Preliminary results of the first 28 patients recruited (11 P-NETs, 12 non-pancreatic carcinoids, 2 medullary thyroid carcinomas and 3 pituitary adenomas) were presented at ASCO GI in 2014, but the study has not been published to date. An ORR of 33% was reported for carcinoids (30% for typical and 50% for atypical), although their primary tumor site was not specified. A very similar rate was reported for P-NETs (36%) [50]. Therefore, CAPTEM may be considered for patients with highly proliferative, rapidly progressive non-pancreatic NETs and/or those refractory to or not suitable for other treatment options, although available evidence to support its use is weak.

CAPTEM has also been explored in combination with ^177^-Lu-Octreotate in a phase I/II trial that accrued 35 patients with advanced NETs. The overall ORR was 53% [34], including 15% complete responses. Response rates were higher in patients with gastropancreatic NETs (82%) than in those with bowel primaries (26%), while the 2 lung NET patients included only achieved disease stabilization. Based on these results, the AGITG CONTROL NET Study midgut cohort (N = 45) randomized (2:1) patients to receive PRRT and CAPTEM vs PRRT alone. Preliminary results showed numerically higher ORR for the combination (31 vs. 15%) with no clear PFS benefit (PFS rate at 15 months: 92 vs. 90%), and at the expense of greater mielotoxicity. Longer follow-up is needed to assess whether the increased RR is translated or not to a clinically meaningful PFS benefit to justify the increased toxicity associated with the combination [36].

Finally, platinum-based regimens have been particularly assessed in lung NETs given their efficacy in small cell lung NECs. Several small retrospective studies evaluated platinum-etoposide in typical and atypical lung carcinoids and documented ORRs of 23 to 39%; one of them reported a median PFS of 7 months [42, 63–65]. One of these studies, that included 36 patients (15 P-NETs and 21 EP-NETs), reported a superior efficacy of platinum-etoposide in lung or thymus NETs (RR 39%) than in NETs of pancreatic origin (27%) [42]. Among patients with lung NETs treated with XELOX in the prospective trial by Bajetta et al. [43], ORR (60%) seemed superior to that observed in P-NETs, although numbers of patients per subgroup are so small that no firm conclusions may be drawn in this regard. Table 2 summarizes the results of phase III and selected phase II chemotherapy trials in EP-NETS.

**Table 2:** Chemotherapy in EP-NETs: results from phase III and selected phase II trials

Author	Phase	N (GI/L)	Treatment	ORR (%)	mPFS (months)	mOS (months)
Classical regimens based on STZ, DOXO and DTIC						
Moertel et al 1979 [52]	III	118 (50/17)	STZ + 5-FU vs STZ + CTX	33.0 vs 26.0 (GI: 36.4 vs. 37.5/L: 28.6 vs 0.0)	NR	11.2 vs 12.5
Engstrom et al.1984 [53]	II/III	172 (85/18)	5-FU + STZ vs DOXO	22.0 vs 21.0	NR	16.0 vs 12.0 (P = ns)
Sun et al 2005 [54]	II/III	176 (55/22)	DOXO + 5FU vs STZ + 5-FU	15.9 vs 16.0 (P = ns)	4.5 vs 5.3 (P = ns)	15.7 vs 24.3 (P = 0.027)
Sun et al 2005 [54]	II/III	61 (21/11)	DTIC	8.2	NR	11.9
Dahan et al 2009 [55]	III	64 (42/3)	INFα-2A vs STZ + 5FU	9.0 vs 3.0	14.1 vs 7.3 (HR 0.75/P = 0.25)	44.3 vs 30.4 (P = 0.83)
Bokowski et al 1994 [56]	II	56 (28/7)	DTIC	15.0	NR	20.0
Bajetta E. et al 1998 [17]	II	30 (6/3)	DTIC + 5-FU + EPI	30.0 (GI:17.0/L: NR)	Not reached	Not reached
Bajetta E et al. 2002 [16]	II	82 (17/7)	DTIC + 5-FU + EPI	24.4 (L:14.0)	21.0 ^b^	38.0
Meyer et al 2014 [13]	II	86 (17/0)	STZ + CAP vs STZ + CAP + CDDP	12.0 vs 16.0	10.2 vs 9.7 (P = NR/HR 0.74)	26.7 vs 27.5 (HR 1.16/P = NR)
Capecitabine and Temozolomide based combinations						
Ferolla et al. 2020 [61]	II	40 (0/36)	LAN + TEM	2.5	9.2	Not reached
Kulke et al 2006 [20]	II	29 (11^a^)	TEM + Thalidomide	25 (7.0 ^a^)	Not reached	Not reached
Chan JA et al 2012 [21]	II	34 (19 ^a^)	TEM + BEVA	15 (0.0 ^a^)	11.0 (7.3 ^a^)	33.3 (18.8 ^a^)
Mitry et al 2014 [59]	II	49 (49/0)	CAP + BEVA	18.0	23.4	Not reached
Claringbold P.G et al. 2012 [34]	II	35 (15/2)	CAPTEM + 177-Lu-Octreotate	53.0	31.0	Not reached
Fine et al 2014 [50]	II	28 (0/12)	CAPTEM	43.0 (L:41.0)	Not reached	Not reached
Berruti et al 2014 [51]	II	26 (13/8)	CAP + BEVA + OCT	11.5	14.9 (GI:14.3/L: 18.6)	Not reached (L: 38.0)
Pavlakis et al. 2020 [36]	II	47 (47/0)	CAPTEM + PRRT vs. PRRT	31.3 vs 15.4	Not reached	Not reached
Platinum-based regimens						
Moertel et al. 1991 [41]	II	27 (13^a^)	CDDP + VP-16	7.0 (0.0^a^)	NR (3.0^a^)	15.0 (10.5^a^)
Fjällskog ML et al. 2001 [42]	II	36 (3/18)	CDDP + VP-16	36.0 (GI:33.0/L: 39.0)	NR	19.0 (GI:10/L:26.0)
Bajetta et al 2007 [43]	II	27 (8/5)	XELOX	30,0 (GI:0.0/L: 60.0)	20.0 ^b^	40.0
Kunz PL et al 2016 [44]	II	20	XELOX + BEVA	5.0	19.1	42.2
Kunz PL et al 2016 [44]	II	22	FOLFOX + BEVA	18.0	19.3	33.1

*BEVA* Bevacizumab, *CAP* capecitabine, *CAPTEM* capecitabine-temozolomide, *CDDP* cisplatin, *CTX* cyclophosphamide, *DOXO* doxorrubicin, *DTIC* dacarbazine, *EPI* epirubicin, *EVE* everolimus, *FOLFOX* 5-fluorouracile-oxaliplatin, *GI* gastrointestinal, *L* lung, *LAN* lanreotide autogel, *m* months, *mOS* median overall survival, *mPFS* median progression free survival, *NR* not reported, *ns* non signicant, *OCT* octreotide, *ORR* objective response rate, *PRRT* peptide receptor radionuclide therapy, *STZ* streptozocin, *TEM* temozolomide, *VP-16* etoposide, *XELOX* capecitabine-oxaliplatin, *5-FU* 5-fluorouracil

^a^EP-NETs or carcinoids not otherwise specify

^b^*TTP* time to tumor progression

A systematic meta-analysis by Lamarca et al. of 20 studies (one randomized phase III trial, 2 randomized phase II studies, 10 single-arm phase II trials and 7 retrospective analyses) and 264 patients (median of 11 patients per study, range 6–49) suggests a limited role of chemotherapy in EP-NETs and points out the poor quality evidence of chemotherapy studies in this context (evidence level-C due to small patient numbers and heterogeneous populations and treatments) [66]. The mean OR, PFS and OS were 11.5% (95%-CI 5.8–17.2), 16.9 months (95%- CI 3.8–30.04) and 32.2 months (95%-CI 10.4–54.2), respectively. In the trials including both P-NETs and EP-NETs the ORR was lower in EP- than in P-NETs [odds ratio (OR) 0.35 (95% CI 0.18–0.66)] but the significance of this difference was lost when the studies with higher risk of bias were excluded. Well-designed, site-specific prospective studies are greatly needed to properly assess the role of chemotherapy in this setting.

Key Messages

- Chemotherapy has limited efficacy in EP-NETs, and thus its use is not recommended on a routine basis.
- Chemotherapy may only be considered in selected individuals with rapidly progressive advanced EP-NETs upon failure of other more effective therapeutic options including somatostatin analogues, everolimus, PRRT and/or locoregional ablative therapies.
- STZ- or TEM-based regimens are the preferred treatment options when chemotherapy is judged to be indicated in selected patients with EP-NETs. Platinum-based combinations may also be considered, particularly in lung NETs.
- Quality of evidence regarding chemotherapy outcomes assessment in EP-NETs is poor and the performance of site-specific clinical trials are highly encouraged to reliably establish the role, if any, of chemotherapy in each particular setting.

## Role of chemotherapy in neuroendocrine carcinomas

### First-line chemotherapy

Evidence to support treatment recommendations for extra-pulmonary G3 NECs is scarce and derives from limited retrospective series or tumor registries and very few small non-controlled clinical trials [67, 68]. Most clinicians, therefore, treat this entity in analogy to the much more common small cell lung NEC or small cell lung carcinoma (SCLC) due to their histological and clinical resemblance. Poorly differentiated extra-pulmonary NECs have also a very aggressive behavior with a median OS of up to 12 months with best available therapy, and of barely 1 month in patients who only receive supportive care [69]. Treatment with CDDP and etoposide (VP-16) has been the classical approach by analogy with SCLC [67]. The first non-randomized phase II study assessing the efficacy of this regimen in NECs was published in 1991 by Moertel et al. [41]. This study reported a RR of 67% (including 17% of complete responses) with a median OS of 19 months. Neuro- and nephro-toxicity were reported in 24% and 66% of patients, respectively. Bone marrow suppression, alopecia and gastrointestinal toxicity were also frequently encountered adverse events. Thereafter, several small retrospective studies have reported ORRs for this regimen that widely range from 17 to 63% [70–74]. More recent large tumor registries have confirmed the efficacy of CDDP-VP-16 in this setting, although ORRs (28–48%) are generally lower than those reported for SCLC [69, 75–78].

Real world data from the NORDIC study [69], that reported results of 305 patients with G3 NECs from 12 Nordic European hospitals, showed very similar efficacy for the combination of carboplatin versus cisplatin with etoposide in this setting. This supports the use of the carboplatin combination given its overall improved tolerability as it is not associated with renal nor neurotoxicity. The most relevant negative prognostic factors for survival were colorectal primary tumor site, poor performance status (PS) and elevated platelets or lactate dehydrogenase (LDH) levels. This study also documented that NECs with Ki-67 < 55% had a lower response rate to platinum-based therapy (15% versus 42%, P < 0.001), although better survival than patients with Ki-67 ≥ 55% (14 versus 10 months, P < 0.001). Based on this observation alternative chemotherapy regimens are generally recommended for these patients. In the prospective French national registry published in 2017 [75], which included GEP NECs (N = 202) and NECs of unknown origin (N = 51), 69% of the patients received first line chemotherapy, 40% second line and 20% third line. In this study only PS and the number of metastatic sites were identified as independent prognostic features. The Ki-67 cut-off value of 55% and primary site identified by Sorbye et al. were not confirmed as prognostic variables. In the French study PE was the treatment of choice in first line (86%) and was associated with an ORR of 50% and a median OS of 11,6 months. There is no prospective data assessing the carboplatin and etoposide combination in NECs. In the largest retrospective study published in 2019 by Frizziero et al. [76] patients with extra-pulmonary NECs were treated with carboplatin-etoposide as first line (N = 106), second line (N = 16) or third line (N = 1) therapy for advanced disease. Carboplatin-etoposide achieved an ORR of 44,3% in the first line setting and of 23,5% as second- or third-line therapy. The PFS and OS for first and after-first line treatment were 6 and 11,5 months and 4,5 and 12,5 months, respectively. No differences in DCR were observed between intravenous or oral etoposide. As in the French registry, the Ki-67 cut-off value 55% was not a predictive factor for response nor prognostic. Carboplatin and etoposide have been also evaluated in combination with paclitaxel in a phase II trial by Hainsworth et al. [79] that included 78 NECs. The ORR was 53%, including 15% complete responses, and was similar regardless of histology or primary tumor site. The median, 2-year, and 3-year survivals for these patients were 14.5 months, 33%, and 24%, respectively. Toxicity was however significant, with G3-4 neutropenia developed by 82% and hospitalization required in 19% of patients. Carboplatin-etoposide is also being currently explored in the NICE-NEC trial (NCT03980925) in combination with nivolumab in chemonaïve patients with G3 GEP NENs or NENs of unknown origin [80]. The results of this trial are expected for 2022.

Several retrospective studies, mostly from Asia, have assessed the efficacy of first line irinotecan and platinum (IP) in NECs with ORR ranging from 25 to 75% and OS of about 12 months [74, 81–85]. A phase II study of IP in high grade NENs that included 20 patients with extrapulmonary NECs reported an ORR of 58% [86]. A randomized Asian study (NCT03168594) is currently comparing CCDP and irinotecan versus CDDP and VP-16 efficacy in GEP NECs [87]. TEM-based regimens have been barely assessed in chemonaïve patients with high grade NECs and are preferentially considered for high-grade NENs with Ki-67 index in the lower range (21–55%) and/or in those with well differentiated tumors (G3 NETs), although evidence to support this recommendation is not too solid [67, 69]. A phase II randomized trial (NCT02595424) is currently comparing CDDP and VP-16 versus CAPTEM in advanced G3 non-small cell GEP NECs and shall help clarify the relative efficacy of these regimens in this setting [88].

Fluoropyrimidine-based regimens with irinotecan or oxaliplatin are generally reserved for second line therapy although many experts advocate their use upfront in large cell NECs of the GI tract, particularly if they are associated with a non-neuroendocrine component (MINEN). A phase II trial has recently evaluated the combination of capecitabine, oxaliplatin and irinotecan (CAPOXIRI) with bevacizumab as first line therapy in 22 gastrointestinal NEC patients followed by pazopanib and capecitabine as maintenances therapy in responders or carboplatin and etoposide in non-responders [89]. Four-drug combination showed an ORR of 47,5% meeting the primary end-point of the study, although this figure does not substantially differ from historical ORR reported for the combination of platinum-etoposide. The toxicity profile was not negligible with G3-4 hematological and non-hematological encountered in 8 and 34 patients, respectively. Median PFS and OS was superior for responders to CAPOXIRI-BEVA than for non-responders: 18 and 30,5 months vs 5 and 14 months, respectively. These results suggest that the switch maintenance strategy in non-responders does not seem to have a major impact on patients’ outcomes, but it is difficult to interpret what is the added value of pazopanib maintenance in responders versus bevacizumab or fluoropyrimidines alone in this setting. Randomized studies are needed to address whether 3- or 4-drug regimens can significantly improve efficacy or just increase toxicity.

### Second or subsequent lines of therapy

There is not an established second line treatment for NEC patients. Re-challenge with platinum-etoposide may be considered after a break of at least 3–6 months in patients that responded to first line treatment in the absence of significant residual toxicity (neurotoxicity, ototoxicity…). In the NORDIC study the median ORR for the 84 assessable patients that received second line chemotherapy (35 TEM-based and 20 taxotere-based regimens) was 18% and the median survival from start of first-line chemotherapy 19 months. FOLFIRI, FOLFOX or XELOX are currently the most commonly used regimens after platinum-etoposide failure [67]. Retrospective studies showed ORRs in the second-line setting of about 30% and PFS of 4–5 months [90–92]. Second line therapy in the French observational study reported ORR of 24% and 16% with FOLFIRI and FOLFOX, respectively, and a median OS < 6 months [75]. Temozolomide has been also explored in NECs, although its efficacy seems to be lower than in NETs. Whereas a small retrospective study by Weling et al. reported an ORR of 33% with TEM alone or in combination with capecitabine and bevacizumab in 25 NECs [93], no responses were observed with TEM alone in another cohort of 28 NECs [94]. In the phase II trial conducted with TEM in monotherapy in NECs by Kobayashi et al., an ORR of 15% was reported with a median PFS and OS of 1,8 and 7,8 months, respectively [95]. TEM is therefore not generally recommended as single agent for the treatment of NECs. A recent meta-analysis including 19 studies and a total of 582 patients with extra-pulmonary NECs showed limited efficacy of second-line chemotherapy in this malignancy. Global median ORR was 18% (range 0–50), and the median PFS and OS were 2,5 and 7,6 months, respectively [96]. Table 3 summarizes prospective and and selected retrospective studies in NECs.

**Table 3:** Chemotherapy in NECs: Prospective and selected retrospective studies

Author	Type of study	N	Primary site	Treatment	ORR (%)	mPFS (months)	mOS (months)
First line							
Moertel et al. 1991 [41]	II	18	GEP (14)/L (1)/U(3)	CDDP + VP-16	67.0	11.0	19.0
Hainsworth et al. 2006 [79]	II	78	GEP (15)/L (7)/U (48)/ other (8)	Paclitaxel + CBDCA + VP-16	53.0	7.5	14.5
Bajetta et al. 2007 [43]	II	13	GEP (5)/L (5)/ other (3)	XELOX	23.0	4.0 ^b^	5.0
Mani et al. 2008 [86]	II	20	NEC (NR)	CDDP + IRI	58.0	4.0 ^b^	NR
Alifieris et al. 2020 [89]	II	22	GI	CAPOXIRI-BEVA PAZO + CAPE	47.4	13.0	29.0
Walter T et al. 2017 [75]	NTR(RENATEN, FFCD,TENpath)	152	GEP/U	CDDP + VP-16 (113) CBDCA + VP-16 (39)	50.0	6.2	11.6
Mitry et al. 1999 [70]	Retrospective	41	GEP (20)/L (10)/ HN (4)/ U (7)	CDDP + VP-16	41.5	8.9	15.0
Iwasa et al. 2010 [71]	Retrospective	21	P (10)/ HB (11)	CDDP + VP-16	14.0	1.8	5.8
Sorbye et al. 2013 [69]	NTR (NORDIC)	252	GEP (174)/ U (78)	CDDP + VP-16 (129) CBDCA + VP-16 (67) CBDCA + VP-16 + VINC(28)	31.0 30.0 44.0	4.0 4.0 4.0	12.0 11.0 10.0
Yamaguchi, 2014 [74]	Retrospective	206	GEP	CDDP + VP-16 (46) CDDP + IRI (160)	28.0 50.0	4.0 5.2	7.3 13.0
Frizziero et al. 2019 [76]	Retrospective	98	GEP (72)/ U (26)/ other ^a^	CBDCA + VP-16	47.9	6.0	11.5
Jimenez-Fonseca et al. 2020 [78,100]	NTR (RGETNE)	279	GEP (70%) U (16%)	CDDP/CBDCA + VP-16	73.0	6.1	14
Okita et al. 2011 [82]	Retrospective	12	Gastric	CDDP + IRI	75.0	7.0	22.6
Nakano et al. 2012 [84]	Retrospective	28	GEP (9)/ U (12)/ others (23)^a^	CDDP + IRI	64.0	7.3	16.0
Ramella et al. 2013 [81]	Retrospective	28	GEP (19)/ U (6)/ others (2)	CDDP + IRI (25) CBDCA + IRI (3)	46.0	3.7 ^b^	11.7
Lu et al. 2013 [85]	Retrospective	16	GEP	CDDP + IRI	57.1	5.5	10.6
Okuma et al. 2014 [83]	Retrospective	12	Esophageal	CDDP + IRI	50.0	4.0	12.6
Second line and beyond							
Kobayashi et al. 2021 [95]	II	13	GEP (10)/ U (1)/ other (2)	TEM	15.4	1.8	7.8
Walter T et al. 2017 [75]	NTR(RENATEN, FFCD,TENpath)	105	GEP and U	FOLFIRI (72) FOLFOX (33)	24.0 16.0	2.9 2.3	5.9 3.9
Welin T et al. 2011 [93]	Retrospective	25	GEP (17)/ L (3)/ U (5)	TEM ± CAPE ± BVZ	33.0	6.0	22.0
Olsen et al. 2012 [94]	Retrospective	28	GEP (18)/ L (1)/ U (6)	TEM	0.0	2.4	3.5
Hentic et al. 2012 [90]	Retrospective	19	GEP	FOLFIRI	31.0	4.0	18.0
Sorbye et al. 2013 [69]	Retrospective	100	GEP and U	Various (35% TEM, 20% taxanos…)	18.0	NR	19.0
Ferrarotto et al. 2013 [91]	Retrospective	24	GEP (18)/ L (4)/ U (2)	XELOX	29.0	9.8 ^b^	Not reached
Hadoux et al. 2015 [92]	Retrospective	20	GEP (12)/ L (4)/ U (2)/ other (2)	FOLFOX	29.0	4.5	9.9
Yamaguchi, 2014 [74]	Retrospective	116	GEP/ HB	Amrubicin CCDP or CBDCA + VP-16 IRI S-1 CDDP + IRI	4.0 17.0 5.0 27.0 40.0	1.9 1.9 2.2 2.4 4.8	8.0 5.0 6.0 12.0 9.0
Frizziero et al. 2019 [76]	Retrospective	17	GEP/ U/ other ^a^	CBDCA + VP-16	23.5	4.5	12.5

*BEVA* Bevacizumab, *CAPE* capecitabine, *CAPOXIRI* Capecitabine, *Oxaliplatin* Irinotecan, *CBDCA* carboplatin, *CDDP* cisplatin, *FFCD* Fédération Francophonede Cancérologie Digestive, *FOLFIRI* 5-Fluorouracile-irinotecan, *FOLFOX* 5- Fluorouracile-oxaliplatin, *GI* gastrointestinal, *GEP* gastroenteropancreatic, *HB* hepatobiliary, *HN* head and neck, *IP* Irinotecan-platinum, *IRI* Irinotecan, *L* lung, *MANEC* Mixed adenoneuroendocrine carcinoma, *mOS* median overall survival, *mPFS* median progression free survival, NEC neuroendocrine carcinoma, *NR* not reported, *NTR* national tumor registry, *ORR* overall response rate, *OS* overall survival, *PAZO* Pazopanib, *PFS* progression free survival, *RENATEN* Groupe d’étude des Tumeurs Endocrines [GTE], *RGETNE* Registro del grupo español de tumores neuroendocrinos, *TENpath* Réseau national d’expertise pour le diagnostic anatomopathologique des tumeurs neuroendocrines de l’adulte, familiales et sporadiques, *TTP* time to tumor progression, *U* unknown, *VINC* vincristine, *VP-16* etoposide, *XELOX* capecitabine + Oxaliplatin

^a^Data from the entire cohort, no specific of the subgroup

^b^*TTP* time to tumor progression

Several ongoing trials are currently assessing different treatment regimens in the second line setting of high grade NENs. These include the SENECA study, a randomized phase II trial comparing CAPTEM versus FOLFIRI (NCT03387592) [97], the BEVANEC study (NCT02820857), a phase II randomized trial assessing FOLFIRI with or without bevacizumab upon progression to PE [98], and the NET-02 study (NCT03837977), a phase II randomized trial evaluating liposomal irinotecan (Nal-Iri) and 5-FU versus docetaxel as second line therapy in extra-pulmonary NECs [99].

Key messages

- Poorly-differentiated neuroendocrine carcinomas have a very poor prognosis with a median OS of less than 12 months with best available therapy.
- Chemotherapy is an essential part of the multimodality approach for localized NECs and the mainstay of care in advanced disease, although no randomized trials have ever been conducted in G3 extra-pulmonary NENs.
- Platinum-etoposide combinations are the regimens of choice based on retrospective series or tumor registries and a few small non-controlled clinical trials, although RR reported in extra-pulmonary NECs are lower (< 50%) than those reported for SCLC. Carboplatin combinations are preferred over cisplatin ones as they seem to have similar efficacy, while the tolerance is better.
- Second line chemotherapy is not routinely recommended in NECs. Fluoropyrimidine-based chemotherapy, such as FOLFOX, FOLFIRI or CAPTEM, may be considered in subsequent lines of therapy in patients with good PS after careful discussion of potential risks and benefits with the patient.
- TEM- or STZ-based regimens are generally preferred for well-differentiated high grade neuroendocrine tumors (G3 NETs). Nevertheless, evidence to support this recommendation is scarce and efficacy of these regimens in this subset of patients shall be prospectively assessed.

## Treatment algorithm: when is chemotherapy indicated?

### Indication of chemotherapy in NETs

- **Adjuvant treatment**
- Adjuvant treatment is not recommended in G1-2 GEP-NETs as recurrence rates are generally low and there are no data to support postoperative therapy is of any value in this context [68, 101, 102, 110]. Large retrospective studies have reported no benefit of adjuvant therapy in neither typical nor atypical lung carcinoids [105–110].
- Therefore, adjuvant therapy is not routinely recommended for lung NETs [101–104]. However, adjuvant chemotherapy (platinum-based or temozolomide-based regimens), with or without radiotherapy, may be considered in selected fit patients with particularly high risk of relapse (i.e. N2 atypical carcinoids with high proliferation index) after multidisciplinary discussion [102, 105, 106].
- For thymic carcinoids, evidence is even poorer [111–114].
- Case-by-case discussion is recommended to decide additional local and/or systemic treatment options in thymic carcinoids following R0 (if stage 3 or 4) or R1 or R2 resection [104].
- **Metastatic disease**
- Systemic chemotherapy is primarily indicated in advanced progressive G1-2 NETs of pancreatic origin. Chemotherapy may be considered upfront in P-NETs with bulky disease also in the absence of documented prior disease progression, particularly in tumors with Ki-67 index above 10% [68, 101, 110, 111]. STZ-based chemotherapy has been the standard of care in P-NETs for many years, although the CAPTEM combination is being increasingly used since results from the ECOG-2211 trial suggest similar activity to the older regimen, and it is associated with improved tolerability and patients’ convenience as it is orally administered. Both regimens are valid treatment options for P-NETs when chemotherapy is indicated, and are currently being compared head-to-head in the BETTER-2 trial. The optimal integration of chemotherapy with other treatment options in this setting is a matter of debate, and some ongoing trials (i.e. SEQTOR, COMPETE,..) shall provide relevant information in the near future to help clinicians optimize the sequential use of available treatment options in P-NETs. Regarding EP-NETs, efficacy of cytotoxic chemotherapy is rather limited and thus its use cannot be recommended on a routine basis. Chemotherapy may only be considered in selected individuals with rapidly progressive tumors upon failure of other more effective therapeutic options including somatostatin analogues, everolimus and/or PRRT [68, 102, 115, 116]. There is no solid data to support any particular cytotoxic regimen in this setting, although the most commonly used are STZ-based, TEM-based or platinum-based regimens. Therefore, enrollment of patients in clinical trials is highly encouraged in this context whenever available.

### Indication of chemotherapy in NECs

- **Adjuvant treatment**
- Chemotherapy is an essential part of the multimodality approach for localized NECs and the mainstay of care in advanced disease, although no randomized trials have ever been conducted in G3 extra-pulmonary NENs. Based on the high risk of systemic relapse after primary tumor resection, all experts and guidelines agree to recommend adjuvant systemic platinum-based chemotherapy following surgery in patients with localized NECs [67, 68, 101, 104, 116]. Cisplatin or carboplatin and etoposide for 4 to 6 cycles are generally recommended. Chemotherapy is also indicated in combination with radiotherapy for localized disease when surgery is not feasible or too morbid (i.e. esophageal primary).
- **Metastatic disease**
- Systemic chemotherapy is indicated in patients with advanced unresectable disease and adequate performance status and organ function. Otherwise, patients may be just offered best supportive care. The combinations of cisplatin or carboplatin with etoposide are the most widely used in NECs [67, 68, 101, 104, 116]. Carboplatin is generally preferred over cisplatin as it has similar antitumor activity and better toxicity profile [69]. Alternative regimenssubstituting irinotecan for etoposide are also acceptable first line options, more commonly used in Asia [74]. Although second-line regimens have not been evaluated rigorously either, the most widely accepted options include temozolomide-, irinotecan- or oxaliplatin-based schedules.
- The optimal chemotherapy regimen for the recently recognized NET G3 entity is a matter of debate and has not been widely studied yet. G3 NETs generally have a Ki-67 in the lower G3 range (21-50%) and a molecular profile that resembles that encountered in low grade NETs (mutations in DAXX/ATRX, MEN1 or mTOR pathway genes). They have better prognosis than G3 NECs although tumor response rates to platinum agents have been reported to be lower [69]. For these reasons most experts recommend to treat them as high-G2 NETs. Chemotherapy is the first choice of therapy in these patients but TEM- or STZ-based regimens are generally preferred. Nevertheless, prospective assessment of the efficacy of these regimens in this subset of patients is required and currently ongoing.

## Future perspectives

Systemic therapies for NENs have considerably expanded over the past years, although options are still rather limited. Chemotherapy remains an essential component of the treatment strategy of patients with NENs, particularly for those with bulky, symptomatic or rapidly progressive tumors (generally G3 or high-G2 NENs). In the context of NETs, chemotherapy has a well-established role in the management of those of pancreatic origin, whereas its use in L- or GI-NETs is still debated. More recently developed targeted agents (sunitinib, surufatinib, everolimus) have unquestionable advantages, as they have been more adequately assessed versus best supportive care in large, well designed double-blind placebo-controlled randomized trials, and they are orally available thereby improving patient’s convenience. However, as opposed to chemotherapy, these novel targeted agents rarely induce tumor shrinkage, which is a relevant treatment goal in certain subgroups of patients, such as those with bulky, symptomatic disease or borderline-resectable locally advanced tumors. Nevertheless, randomized studies comparing the efficacy and safety of chemotherapy versus other treatment options such as targeted agents, locoregional ablative therapies or PRRT are certainly needed to properly position chemotherapy within the treatment algorithm of NENs. Further research is also needed to explore its efficacy when combined with other agents (mTOR inhibitors, tyrosine-kinase inhibitors, PRRT…) that act through different pathways, particularly those with no overlapping toxicities.

But the major challenge ahead is to identify reliable predictive biomarkers that can help to more adequately select patients most likely to benefit from specific therapies, allowing to move from a “one-size-fits-all” towards a more personalized medicine. Traditional chemotherapeutic agents are cytotoxic by means of interfering with cell division (mitosis) or inducing DNA damage. Therefore, they are particularly toxic to rapidly dividing cells or cells with defects in DNA repair mechanisms. Consistent with this, a higher Ki-67 index or mitotic rate have been associated with increased response to cytotoxic agents, although robust data is lacking [117]. Moreover, both Rb loss and KRAS mutations have been described as predictors of response to platinum-based chemotherapy in G3 P-NENs. [118]. Other molecular alterations associated with platinum-sensitivity in other tumor types include p53 or BRCA mutations; the latter also confer sensitivity to PARP inhibitors [119], although this has not been explored in NENs to date. MGMT (O6-methylguanine DNA methyltransferase) methylation has demonstrated to predict efficacy of alkylating agents in glioblastoma multiforme [120]. MGMT deficiency has been globally related with a trend to a better RR, PFS and OS in NETs treated with alkylating agents (TEM, DTIC) in retrospective studies. Indeed, depletion of MGMT induced by capecitabine has been suggested as the rational for the CAPTEM synergy observed in NETs. Nevertheless, results are inconsistent among different studies maybe due to their heterogeneity (multiple sites of origin, different techniques to asses MGMT status, etc.) and need to be confirmed in prospective trials. [121].

Regarding NECs the results of several ongoing trials will provide very valuable quality data of the efficacy of different chemotherapy regimens in poorly differentiated NECs, both in chemonaïve and refractory carcinomas, and also in high grade well differentiated tumors (G3 NETs) [118]. Combination strategies with immunotherapy and PRRT are also being explored to potentially improve the poor outcomes of chemotherapy in NECs. It should also be noted that at present large and small cell NECs are generally treated in a similar way, although growing evidence suggests these entities differ from a molecular perspective, and this may translate into relevant differences in treatment outcome and prognosis. Small cell morphology is predominant in lung NECs whereas large cell is more commonly encountered in digestive NECs except for esophageal and anal canal primaries. Small cell NECs (SC-NECs) are molecularly more homogeneous and often characterized by bi-allelic inactivation of TP53 and RB1. Large cell NECs (LC-NECs) have a better prognosis than SC-NECs, and within LC-NECs, those of GI origin have a better prognosis than lung LC-NECs [122]. LC-NECs are molecularly more complex and heterogeneous, and up to 40% have a non-neuroendocrine component. At least 2 distinct molecular subtypes have been described in LC-NECs, a “small cell-like signature” (with a lower proportion of TP53 and RB1 mutations observed in extrapulmonary NECs than in lung NECs) and a “carcinoma-like signature” that resembles the molecular profile of the non-neuroendocrine tumors of similar anatomic site (i.e. KRAS mutations in pancreatic NECs, KRAS/BRAF/APC/TP53/MYC mutations in colorectal NECs) [123]. Despite these notable differences, however, clinical guidelines do not recommend to treat NECs differently according to the morphological or molecular subtype, basically due to the lack of data in this regard. Improved international collaboration is therefore urgently needed from the bench to the bedside in order to improve the clinical management and outcome of these patients. Large, well designed prospective clinical trials shall be encouraged to generate good quality data that is particularly needed in this clinical setting. Moreover, personalized treatment options shall be further explored in certain molecular subgroups, such as NECs harboring BRAF mutations, ALK, ROS1 or NTRK traslocations, high TMB or MSI [124–126]. Indeed, there are several drugs currently approved for molecularly-defined, tumor-agnostic indications, such as the immune check-point inhibitor pembrolizumab for the treatment of high TMB or MSI tumors, or the NTRK inhibitors larotrectinib or entrectinib for tumors harboring NTRK, ALK or ROS1 traslocations [127–130]. A deeper understanding of the molecular basis of NEN genesis and progression will be key to improve treatment efficacy and prognosis of these patients.

## Abbreviations

5-FU: 5- Fluorouracil
AEs: Adverse events
BEVA: Bevacizumab
BSC: Best supportive care
CAP: Capecitabine
CAPOX: Capecitabine and oxaliplatin
CAPOXIRI: Capecitabine, oxaliplatin and irinotecan
CAPTEM: Capecitabine and temozolomide
CBDCA: Carboplatin
CDDP: Cisplatin
CTX: Cyclophosphamide
CTZ: Chlorozotocin
CYP450: Cytochrome P450
DCR: Disease control rate
DOXO: Doxorubicin
DTIC: Dacarbazine
ECOG: Eastern Cooperative Group
EP-NETs: Extra-pancreatic neuroendocrine tumors
EPI: Epirubicin
EVE: Everolimus
EVO: Evofosfamide
FFCD: Fédération Francophonede Cancérologie Digestive
FOLFIRI: 5-FU and Irinotecan
FOLFOX: 5-FU and oxaliplatin
GEMOX: Gemcitabine and oxaliplatin
GEP: Gastroenteropancreatic
GI: Gastrointestinal
HB: Hepatobiliary
HN: Head and neck
IP: Irinotecan and platinum
IRI: Irinotecan
L: Lung
LAN: Lanreotide
LDH: Lactate dehydrogenase
m: Months
MANEC: Mixed adenoneuroendocrine carcinoma
MGMT: O-6-methylguanine-DNA methyltransferase
MINEN: Mixed neuroendocrine non-neuroendocrine neoplasia
MSI: Microsatellite instability
MTIC: 3-Methyl-(triazen-1-yl)imidazole-4-carboxamide
NECs: Neuroendocrine carcinomas
NENs: Neuroendocrine neoplasias
NETs: Neuroendocrine tumors
NR: Not reported
Ns: Non significant
NTR: National tumor registry
NTRK: Neurotrophic receptor tyrosine kinase 1
OCT: Octreotide
OR: Odds ratio
ORR: Objective response rate
OS: Overall survival
P-NETs: Pancreatic neuroendocrine tumors
PARP: Poly(ADP)-ribose polymerase
PAZO: Pazopanib
PDGFR: Platelet derived growth factor receptor
PFS: Progression-free survival
PRRT: Peptide receptor radionuclide therapy
PS: Performance status
Rb: Retinoblastoma
RENATEN: Groupe d’étude des Tumeurs Endocrines [GTE]
RGETNE: Registro del grupo español de tumores neuroendocrinos
RR: Response rate
SCLC: Small cell lung cáncer
STZ: Streptozocin
SUN: Sunitinib
TEM: Temozolomide
TMB: Tumor mutational burden
TTP: Time to tumor progression
U: Unknown
VEGF: Vascular endothelial growth factor
VINC: Vincristine
VP-16: Etoposide
XELOX: Capecitabine and oxaliplatin
y: Year
