# Correction to: Chemotherapy in NEN: still has a role?

**DOI:** 10.1007/s11154-022-09741-w

**Published:** 2022-07-01

**Authors:** Paula Espinosa-Olarte, Anna La Salvia, Maria C. Riesco-Martinez, Beatriz Anton-Pascual, Rocio Garcia-Carbonero

**Affiliations:** Oncology Department, Hospital Universitario, 12 de Octubre, Imas12, UCM, Madrid, Spain

## Correction to: Rev Endocr Metab Disord (2021) 22:595–614 https://doi.org/10.1007/s11154-021-09638-0

In the recently published paper, the authors have noticed that it has many mistakes and that it does not correlate with the last reviewed version of the proofs of the manuscript that was sent.

Below are the errors of this paper:

- First column of table 3 entitled “ Chemotherapy in NECs: Prospective and selected retrospective studies” is completely wrong. It has nothing to do with the last version of the proofs. Thereby the subsequent columns in the table do not refer to the article referred to in column 1.
- In Table 2 “Chemotherapy in EP-NETs:results from phase III and selected phase II trials”, reference 67 related to Ferrolla et al. must be reference 61.
- As we specified in the query details of the proofs since reference 67 was changed for 61, the subsequent reference numbers needed to be move upwards, it has not been made. Thereby all the numbers in the “reference section” do not correlate with the text (which do not need to be changed).
- Reference 62 in the published article “efficacy and safety of Lanreotide ATG 120 mg….” was deleted from the final version, but it is in the published paper. It has to be removed.
- Some spelling or bibliographic format errors were identified and modified in the proofs but changes have not been made in the published version. See references 120,121,122 and 124 in the attached document "APRIL 22", that correlate with 121,122,123,125 in the published version. Also, reference 100 “Jimenez-Fonseca” et al. “ reference 101 in the published version” the website "clinicaltrials.gov" has been added at the end, and actually it does not correlate with the cited poster.
- In page 602, in reference “5159” related to “but its combination with bevacizumab or bevacizumab and octreotide in the BETTER and XELBEVOC prospective trials, demonstrated an ORR of 12–18%” there is a punctuation error and it should be “51,59”
- In page 602, reference 63 related to “Two small retrospective series that included 20–33 patients with lung NETs treated with CAPTEM reporte dan ORR of 18–30%, a median PFS of 9–13 months and a median OS of 30–68 months (63)” should be 62 (as it is in the proofs).
- In page 607, reference “105–106” related to “Large retrospective studies have reported no benefit of adjuvant therapy inneither typical nor atypical lung carcinoids (105–106)” (in 3.1 Indication of chemotherapy in NETs, first pharagraph: adjuvant treatment), is wrong. The correct citation is “105–110” as it is in the proofs.
- In page 608, reference “68,102,115,106” related to “ Chemotherapy may only be considered in selected individuals with rapidly progressive tumor upon failure to other more effective therapeutic options including somatostatin analogues, everoimus and/or PRRT” (in 3.1 Indication of chemotherapy in NETs, metastatic disease), is wrong. The correct citation is “68,102,115,116” as it is in the proofs.

The original article has been corrected.

